# Supplementary material for: Asperphenyltones A and B: New Phenylfuropyridinone Skeleton from an Endophytic Aspergillus sp. GXNU-A1
Source: Molecules. 2022 Nov 23;27(23):8160. doi: 10.3390/molecules27238160 (PMC9737011; doi:10.3390/molecules27238160)
Supplement: Supplementary file 1 [file molecules-27-08160-s001.zip › molecules-2026804-supplementary (1).pdf]

SUPPLEMENTARY MATERIAL

Asperphenyltones A and B: Enantiomers with the Phenylfuropyridinone derivatives from an Endophytic *Aspergillus* sp. GXNU-A1

Jiguo Huang [1,†], Xianglong Bo [2,†], Furong Wu [2], Meijing Tan [2], Youquan Wei [2], Lixia Wang [1], Junqiang Zhou [1], Guiming Wu [1] and Xishan Huang [2,*]

[1] School of Chemical Engineering and Technology, Guangdong Industry Polytechnic, Guangdong Engineering Technical Research Center for Green Household Chemicals, Guangzhou, China
[2] State Key Laboratory for Chemistry and Molecular Engineering of Medicinal Resources, College of Chemistry and Pharmaceutical Sciences, Guangxi Normal University, Guilin, China;
* Correspondence: huangxishan13@foxmail.com; Tel.: +86-773-2120958
† These authors contributed equally to this work.

ABSTRACT: Purification of an extract from the mangrove endophytic fungus *Aspergillus* sp. GXNU-A1 resulted in the isolation of a new pair of enantiomers, asperphenyltones A and B (±**1**), together with four known metabolites **2-5**. The structures of asperphenyltones A and B were established by HR-ESI-MS, 1D and 2D NMR data, and further confirmed by single-crystal X-ray diffraction analysis. Compounds **1-5** were evaluated for their anti-inflammatory effects on production of the nitric oxide (NO), and **1**, **3**, and **4** showed weak inhibitory activities against NO production in activated macrophages with IC_{50} values ranging from 26.14 to 40.06 μM, respectively.

KEYWORDS: *Aspergillus* sp.; mangrove endophytic fungus; asperphenyltone A; anti-inflammatory effects

List of supporting information

Figure S1. ^{1}H NMR (400 MHz, DMSO-d_6) spectrum of compound **1**

Figure S2. ^{13}C NMR (100 MHz, DMSO-d_6) spectrum of compound **1**

Figure S3. Heteronuclear Multiple Quantum Coherence (HMQC) (DMSO-d_6) spectrum of compound **1**

Figure S4. ^{1}H-^{1}HCOSY (400 MHz, DMSO-d_6) spectrum of compound **1**

Figure S5. Distortionless Enhanced Polarization Transfer (DEPT) (DMSO-d_6) spectrum of compound **1**

Figure S6. HMBC (DMSO-d_6) spectrum of compound **1**

Figure S7. HR-ESI-MS spectrum of compound **1**

Figure S8. UV spectrum of compound **1**

Figure S9. IR spectrum of compound **1**

Figure S10. ^{1}H NMR (400 MHz, DMSO-d_6) spectrum of compound **2**

Figure S11. ^{13}C NMR (100 MHz, DMSO-d_6) spectrum of compound **2**

Figure S12. ^{1}H NMR (400 MHz, DMSO-d_6) spectrum of compound **3**

Figure S13. ^{13}C NMR (100 MHz, DMSO-d_6) spectrum of compound **3**

Figure S14. ^{1}H NMR (100 MHz, methanol-d_4) spectrum of compound **4**

Figure S15. ^{13}C NMR (100 MHz, methanol-d_4) spectrum of compound **4**

Figure S16. ^{1}H NMR (100 MHz, methanol-d_4) spectrum of compound **5**

Figure S17. ^{13}C NMR (100 MHz, methanol-d_4) spectrum of compound **5**

Table S1–6. Crystal data for compound **1**.

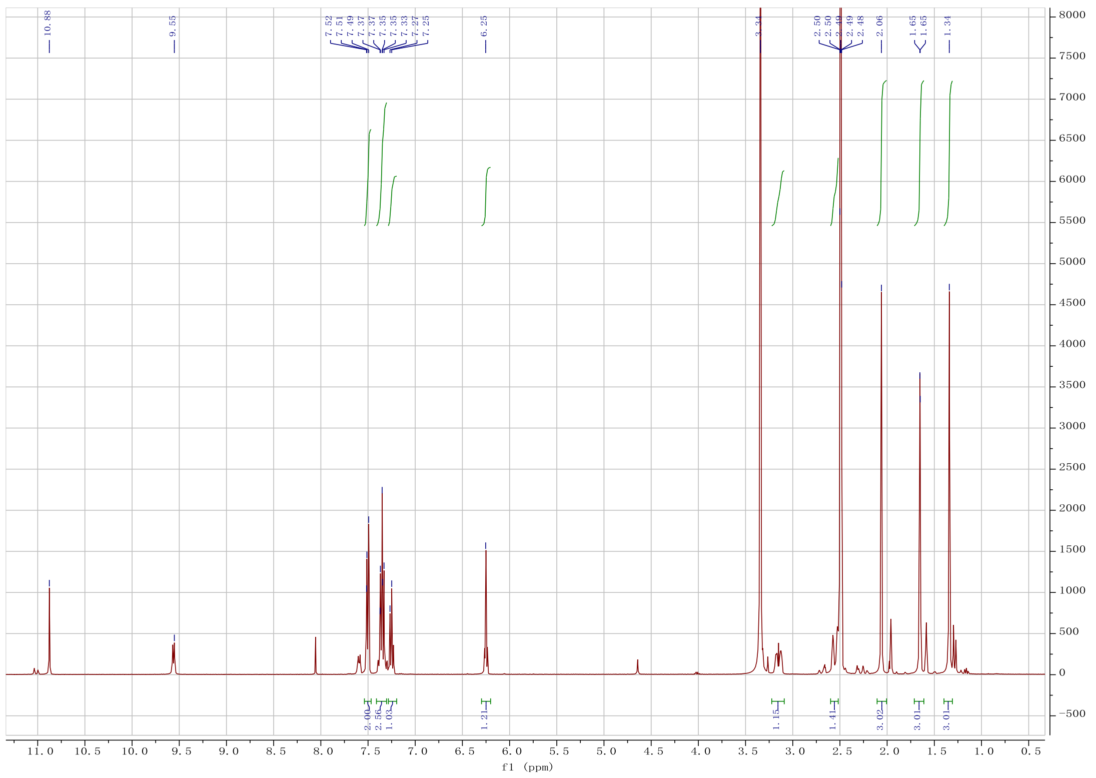

Figure S1. ^{1}H NMR (400 MHz, DMSO-d_6) spectrum of compound **1**

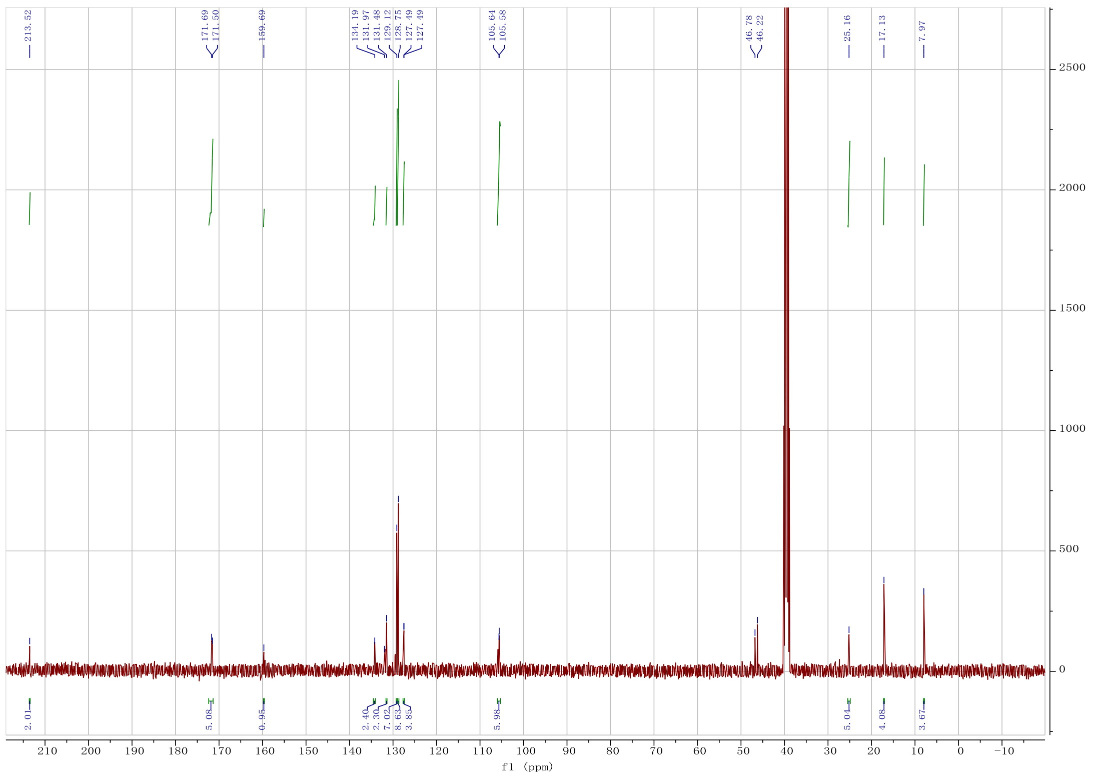

Figure S2. ^{13}C NMR (100 MHz, DMSO-d_6) spectrum of compound **1**

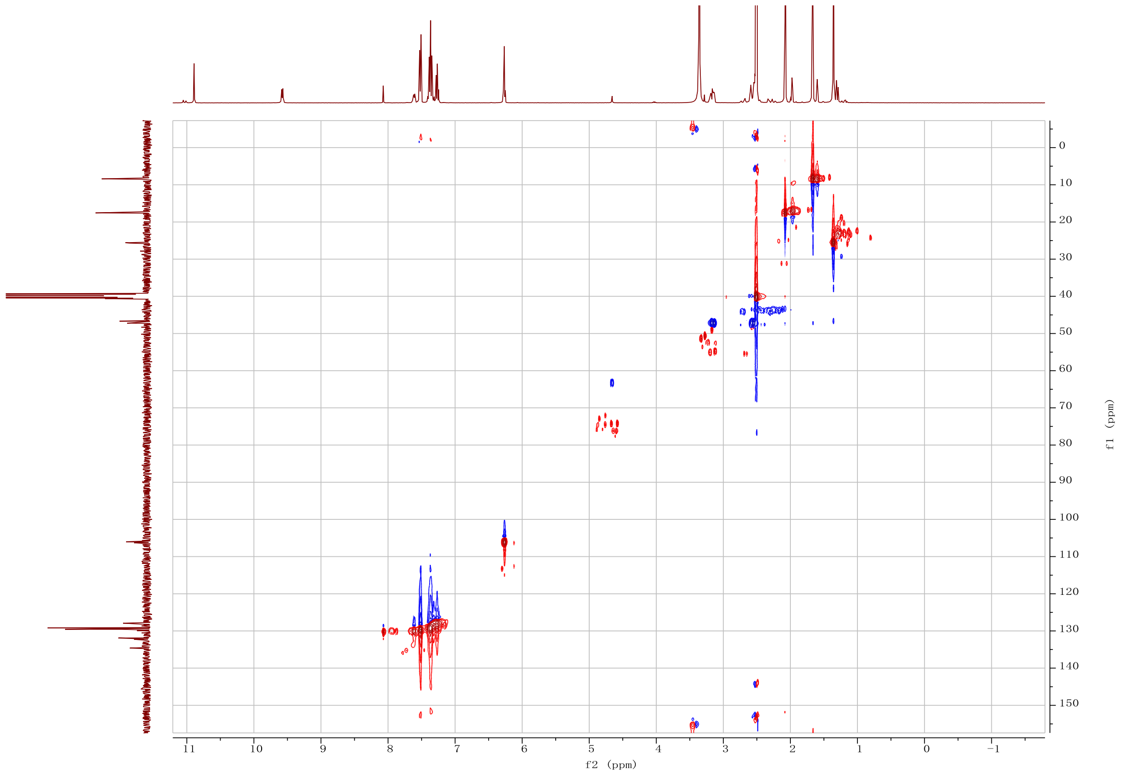

Figure S3. HMQC (400 MHz, DMSO-d_6) spectrum of compound **1**

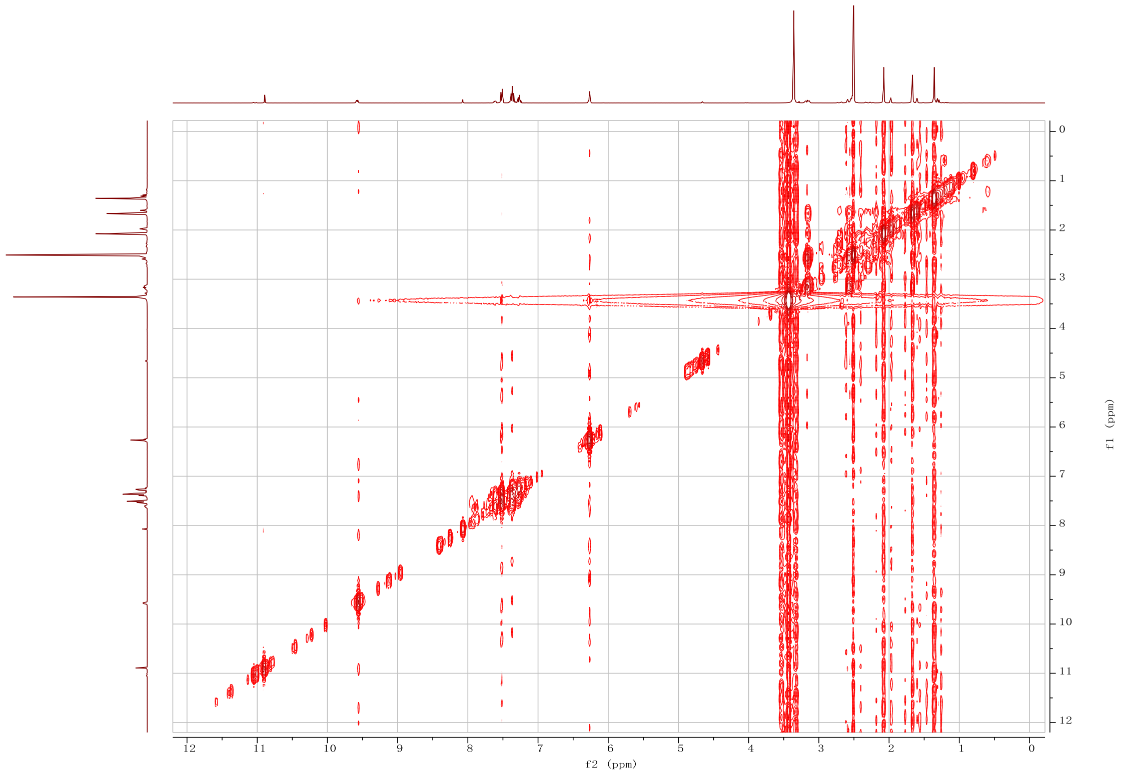

Figure S4. ^{1}H-^{1}HCOSY (400 MHz, DMSO-d_6) spectrum of compound **1**

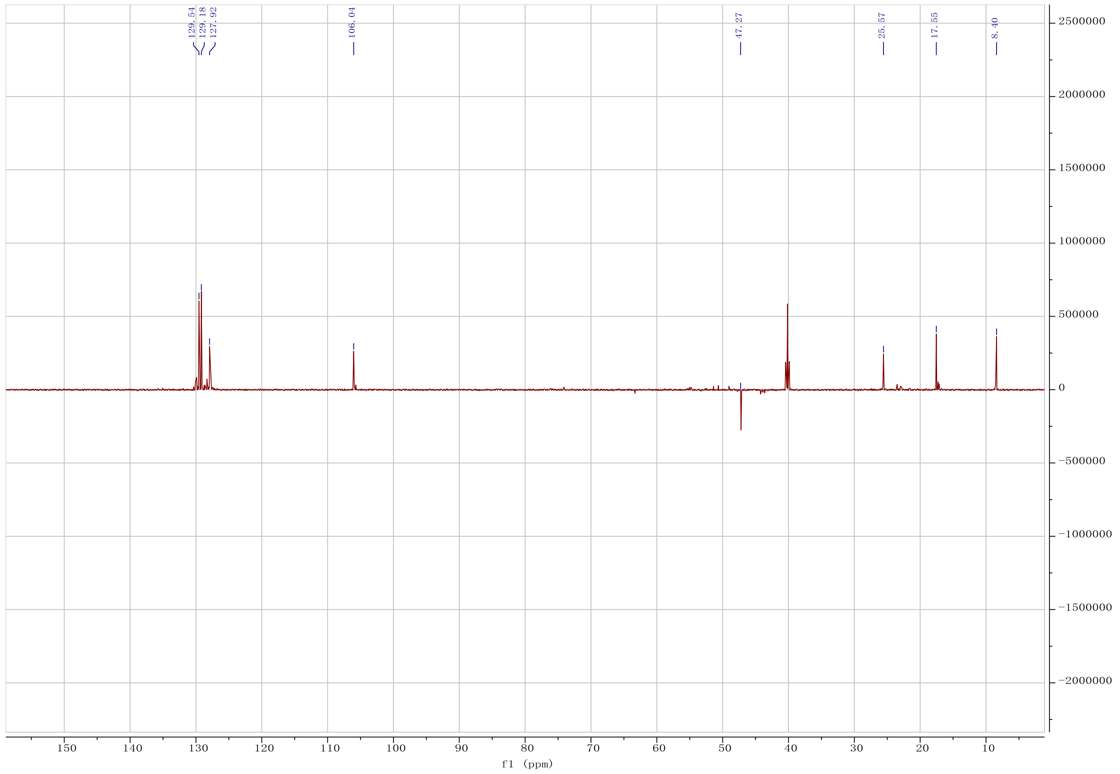

Figure S5. DEPT (400 MHz, DMSO-d_6) spectrum of compound **1**

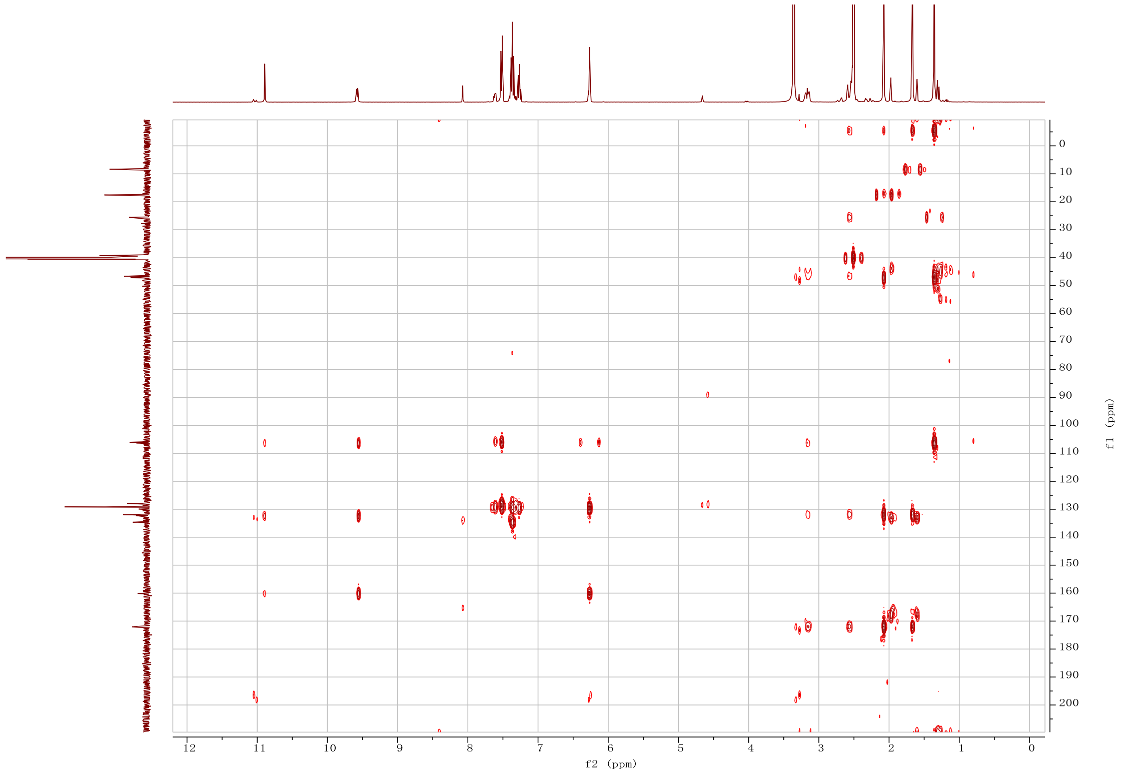

Figure S6. HMBC (400 MHz, DMSO-d_6) spectrum of compound **1**

**样品名称**	H1	**位置**	P1-A2	**仪器名称**	Instrument 1
**用户名称**		**进样体积**	0.5	**进样位置**	
**样品类型**	Sample	**IRM 校正状态**	成功	**数据文件名称**	H2.d
**采集方法**	10-100%YAOJIAN-11-MIN.M	**注释**		**采集时间**	2022/9/7 15:28:48

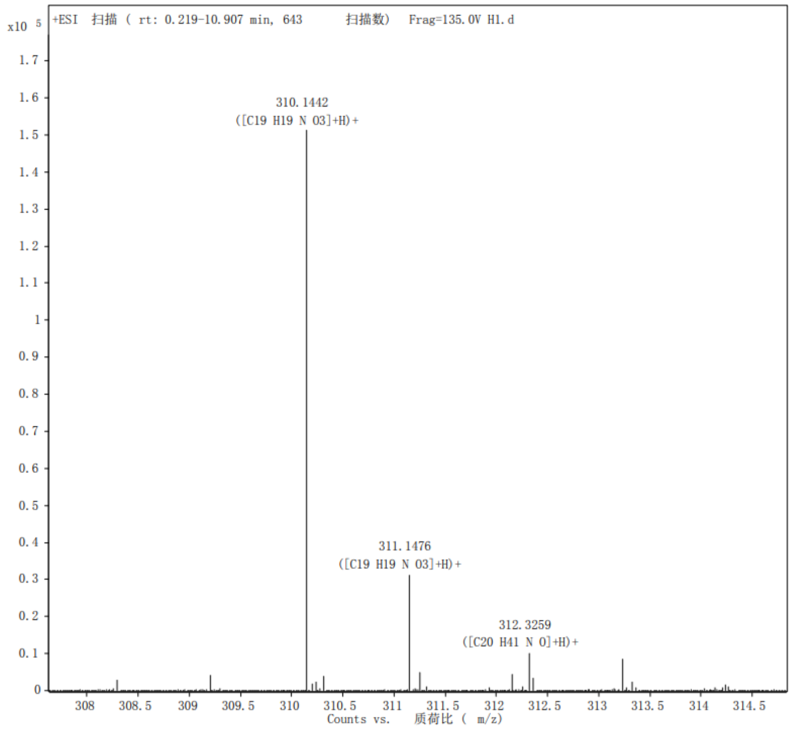

Figure S7. HR-ESI-MS spectrum of compound **1**

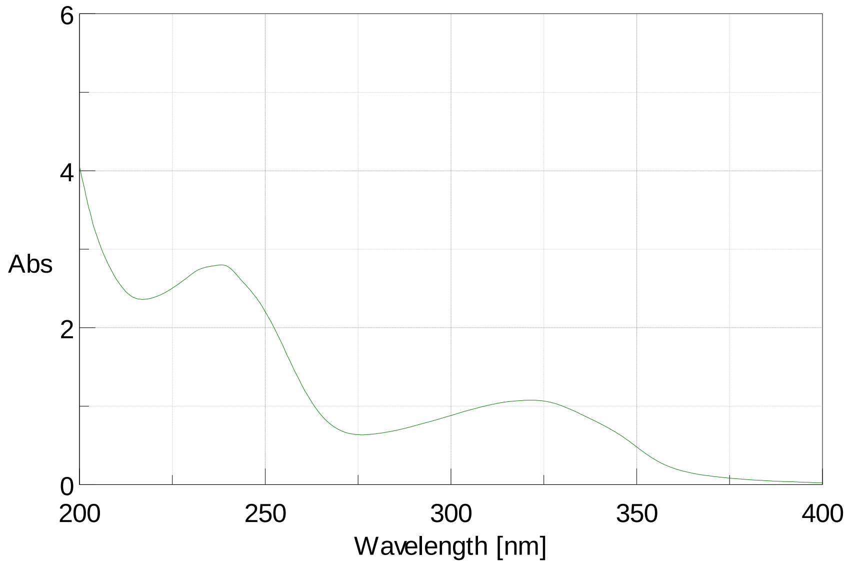

Figure S8. UV spectrum of compound **1**

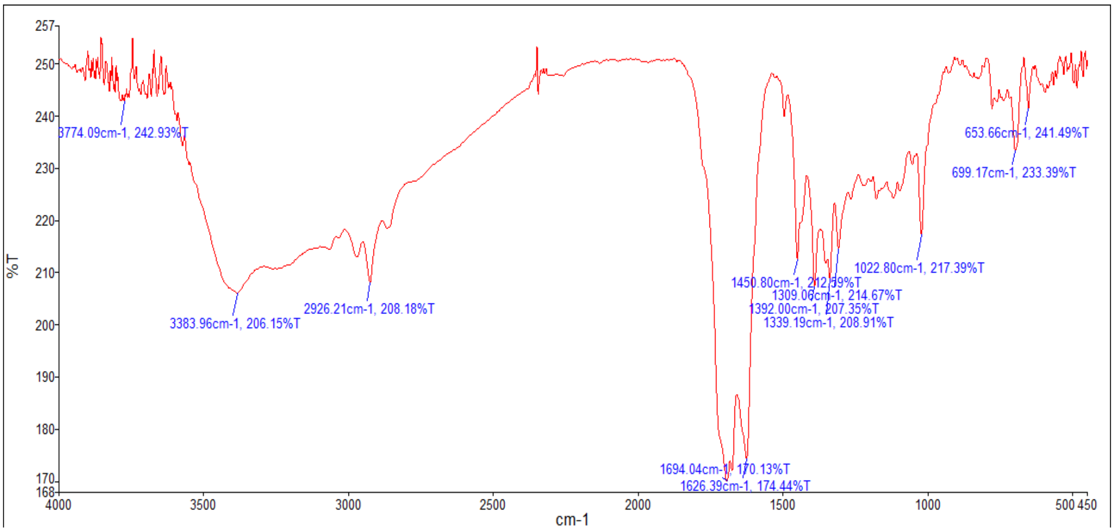

Figure S9. IR spectrum of compound **1**

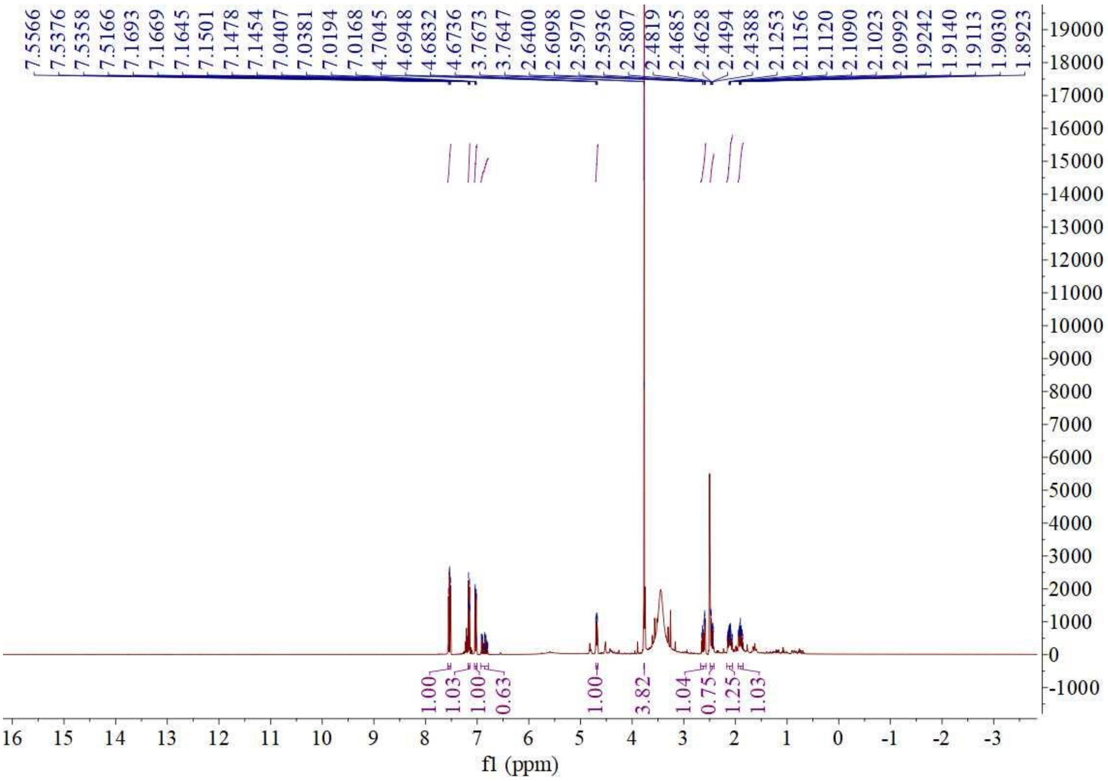

Figure S10. ^{1}H NMR (400 MHz, DMSO-d_6) spectrum of compound **2**

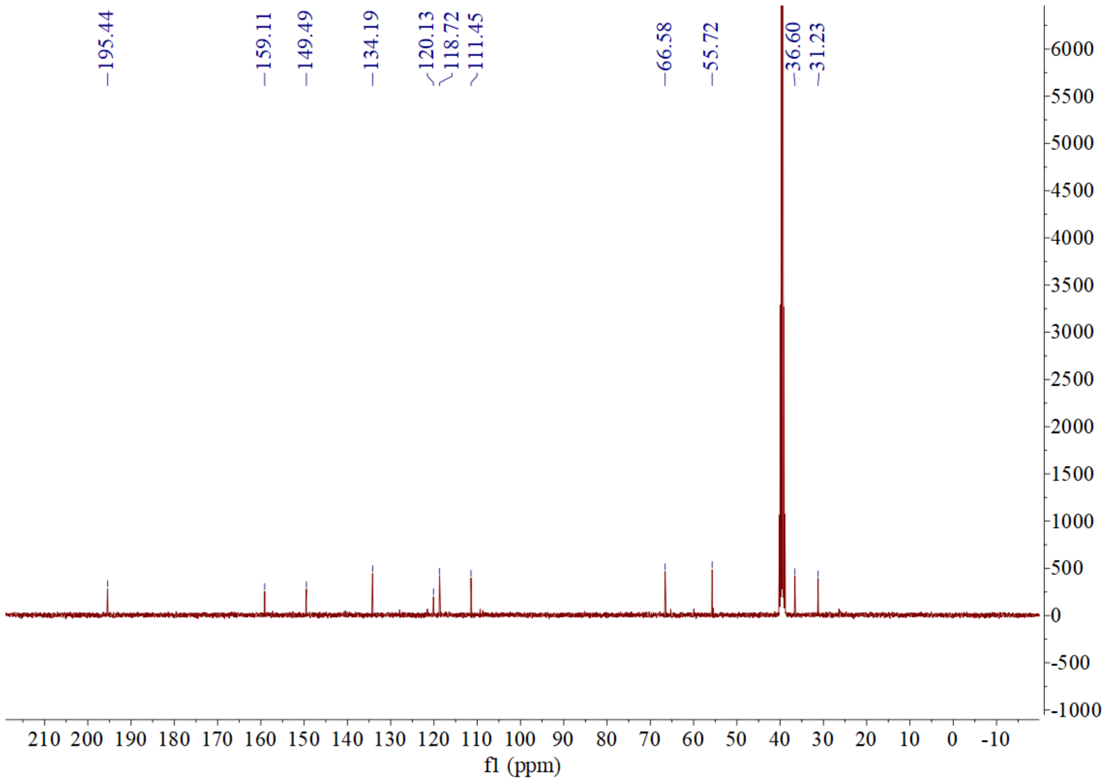

Figure S11. ^{13}C NMR (100 MHz, DMSO-d_6) spectrum of compound **2**

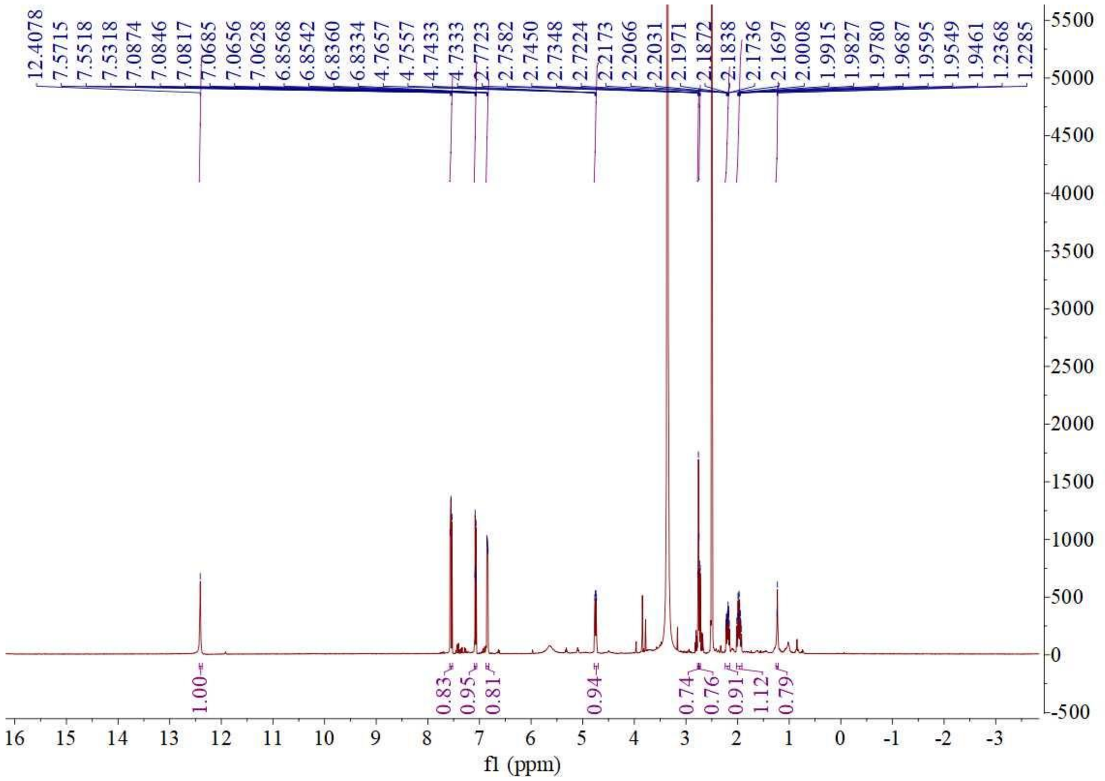

Figure S12. ^{1}H NMR (400 MHz, DMSO-d_6) spectrum of compound **3**

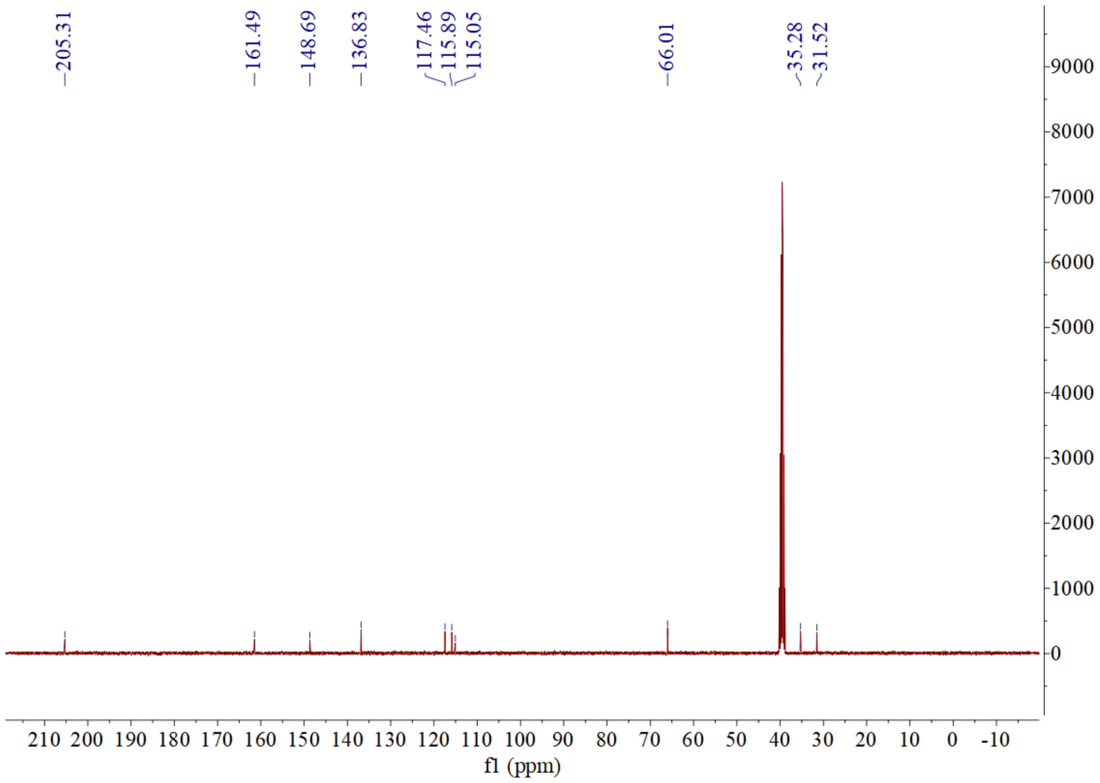

Figure S13. ^{13}C NMR (100 MHz, DMSO-d_6) spectrum of compound **3**

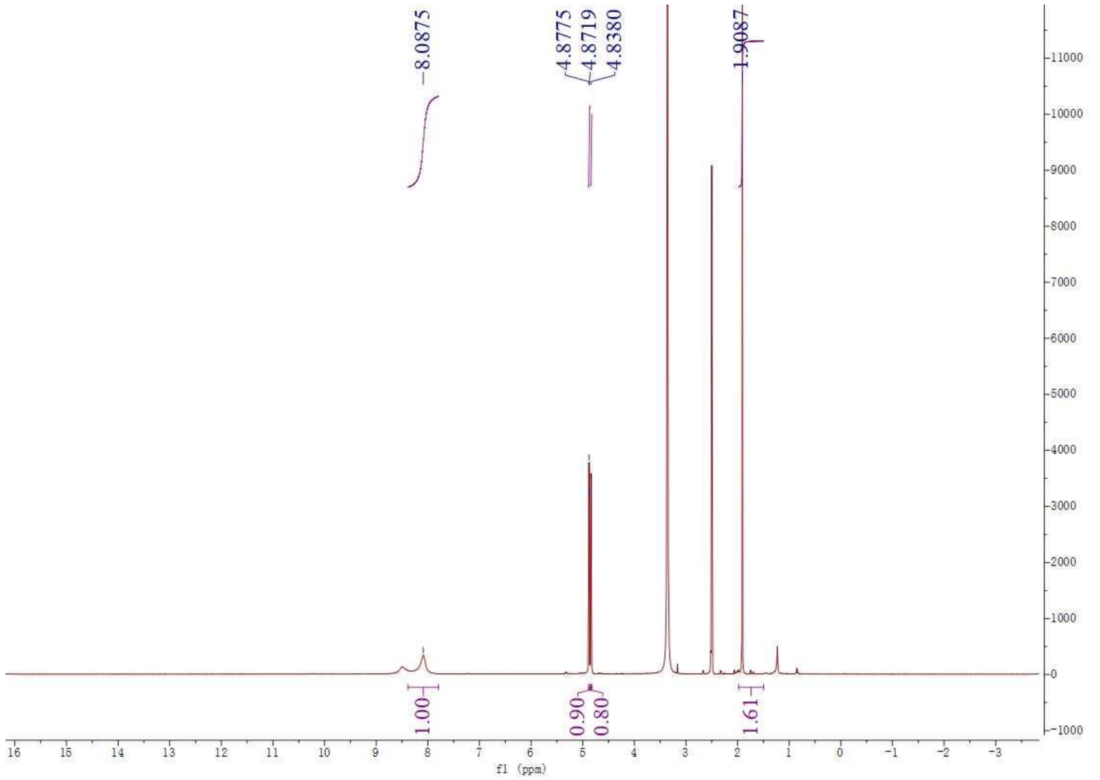

Figure S14. ^{1}H NMR (400 MHz, DMSO-d_6) spectrum of compound **4**

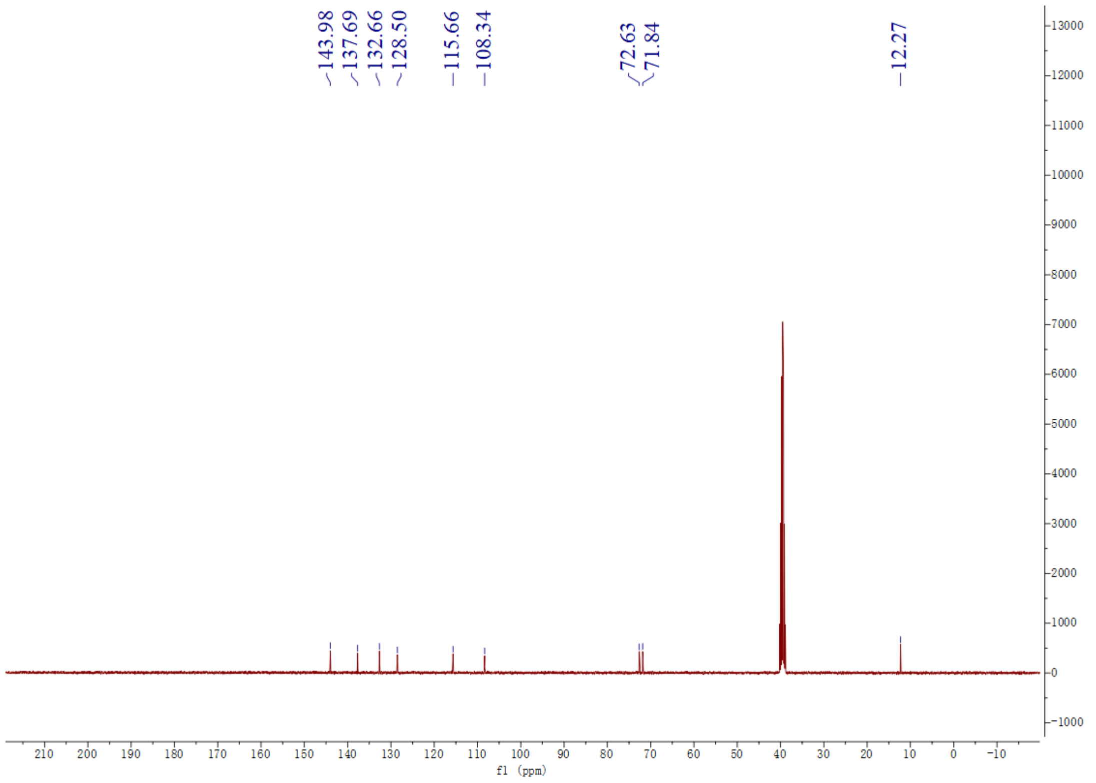

Figure S15. ^{13}C NMR (100 MHz, DMSO-d_6) spectrum of compound **4**

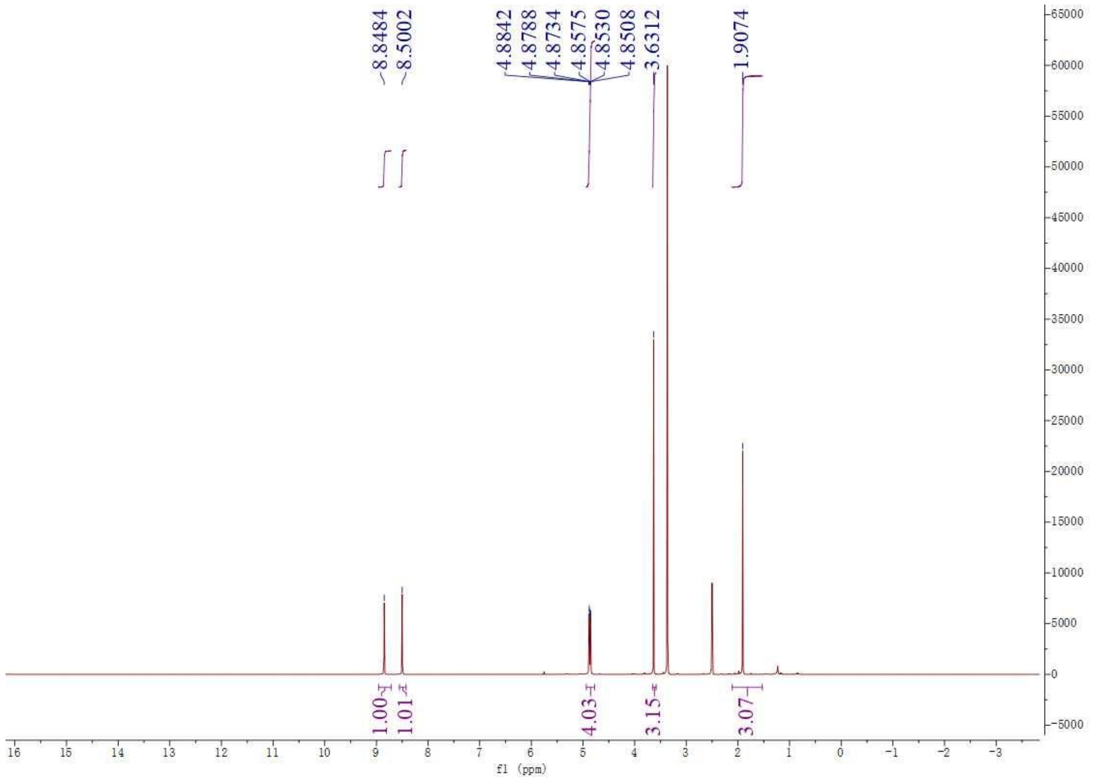

Figure S16. ^{1}H NMR (100 MHz, methanol-d_4) spectrum of compound **5**

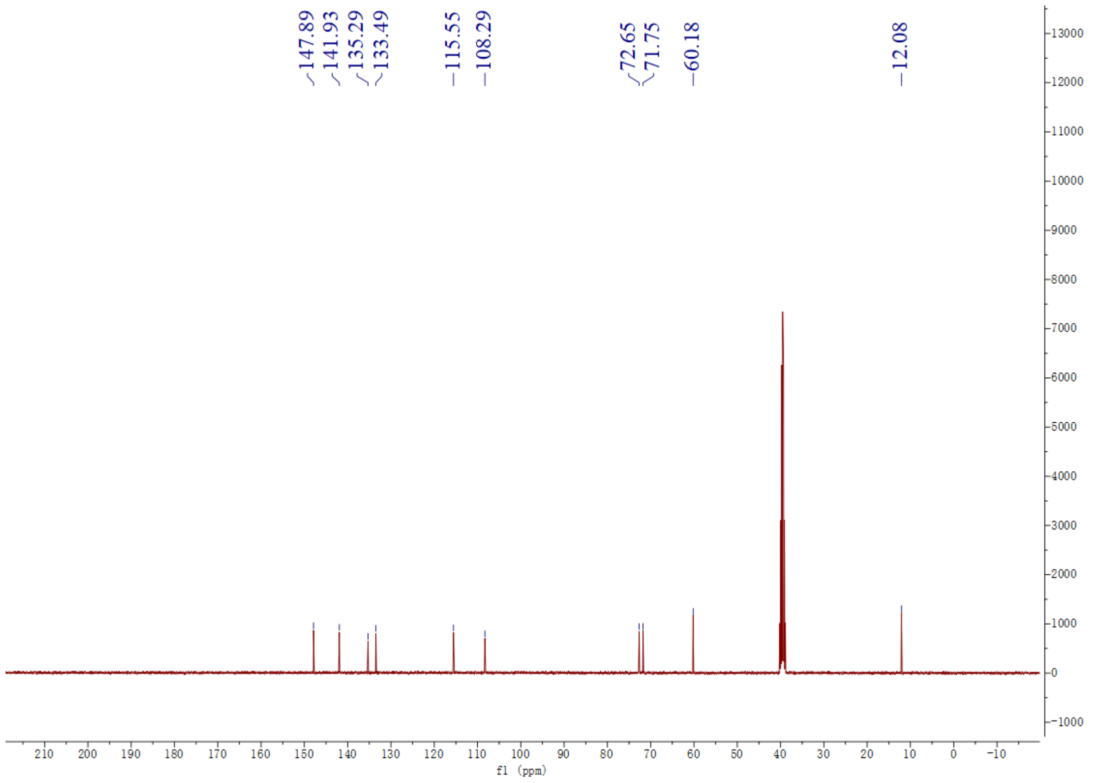

Figure S17. ^{1}C NMR (100 MHz, methanol-d_4) spectrum of compound **5**

Table S1 Crystal data and structure refinement for 1

Identification code	1
Empirical formula	$C_{76}H_{76}N_4O_{12}$
Formula weight	1229.34
Temperature/K	103(6)
Crystal system	monoclinic
Space group	$P2_1/c$
a/Å	9.54190(10)
b/Å	14.6110(3)
c/Å	11.6889(2)
α/°	90
β/°	103.786(2)
γ/°	90
Volume/Å^3	1582.68(5)
Z	1
ρ_{calc}g/cm^3	1.290
μ/mm^{-1}	0.709
F(000)	648.0
Crystal size/mm^3	? × ? × ?

Radiation	CuKα (λ = 1.54184)
2Θ range for data collection/°	9.544 to 151.32
Index ranges	-11 ≤ h ≤ 11, -16 ≤ k ≤ 17, -14 ≤ l ≤ 8
Reflections collected	10965
Independent reflections	3116 [R_{int} = 0.0274, R_{sigma} = 0.0238]
Data/restraints/parameters	3116/0/212
Goodness-of-fit on F^2	1.078
Final R indexes [I>=2σ (I)]	R_1 = 0.0381, wR_2 = 0.0979
Final R indexes [all data]	R_1 = 0.0406, wR_2 = 0.0995
Largest diff. peak/hole / e $Å^{-3}$	0.24/-0.24

Table S2 Fractional Atomic Coordinates (×10^4) and Equivalent Isotropic Displacement Parameters (Å^2×10^3) for compound 1. U_{eq} is defined as 1/3 of of the trace of the orthogonalised U_{IJ} tensor.

Atom	*x*	*y*	*z*	U(eq)
O(001)	8433.1(10)	5541.9(6)	4238.0(7)	19.3(2)
O(002)	4942.4(10)	6092.1(6)	667.3(8)	24.9(2)
O(003)	8192.7(10)	7255.0(6)	4731.4(8)	23.4(2)
N(004)	6495.8(11)	4902.9(7)	1387.7(9)	17.8(2)
C(005)	8568.3(13)	3158.5(8)	2076.5(11)	16.1(3)
C(006)	7589.0(13)	5545.2(8)	3148.6(10)	14.9(2)
C(007)	5930.7(13)	5764.7(8)	1435.4(10)	17.2(3)
C(008)	7567.8(13)	4725.3(8)	2409.4(10)	15.0(2)
C(009)	6686.7(13)	6193.0(8)	2553.6(10)	14.7(2)
C(00A)	7469.8(13)	7614.0(9)	3829.2(10)	17.0(3)
C(00B)	6286.2(13)	7131.8(8)	2908.5(10)	15.7(3)
C(00C)	8460.1(13)	4007.6(8)	2713.3(10)	16.6(3)
C(00D)	7987.9(13)	3040.9(9)	867.5(11)	18.4(3)
C(00E)	7534.9(14)	8574.1(9)	3509.9(11)	19.7(3)
C(00F)	8031.3(14)	2195.2(9)	345.4(11)	21.1(3)

C(00G)	6675.5(15)	8706.4(9)	2429.9(11)	20.8(3)
C(00H)	9269.8(14)	2408.7(9)	2723.3(11)	19.5(3)
C(00I)	4976.0(14)	7058.6(9)	3463.9(12)	22.5(3)
C(00J)	8666.6(14)	1449.4(9)	998.9(12)	22.3(3)
C(00K)	5974.3(15)	7833.8(9)	1894.8(11)	21.4(3)
C(00L)	9306.7(14)	1563.9(9)	2189.7(12)	22.2(3)
C(00M)	8477.6(16)	9242.1(10)	4314.5(13)	29.5(3)
C(00N)	6392.9(19)	9578.3(10)	1752.2(14)	32.3(3)

Table S3 Anisotropic Displacement Parameters ($Å^2 \times 10^3$) for compound 1. The Anisotropic displacement factor exponent takes the form: $-2\pi^2[h^2a^{*2}U_{11}+2hka^*b^*U_{12}+\ldots]$.

Atom	U_{11}	U_{22}	U_{33}	U_{23}	U_{13}	U_{12}
O(001)	25.7(5)	18.1(4)	10.9(4)	-1.7(3)	-2.2(3)	-0.2(4)
O(002)	29.8(5)	20.0(5)	17.9(5)	-5.1(3)	-8.2(4)	5.7(4)
O(003)	26.5(5)	24.0(5)	15.9(4)	-6.3(4)	-2.7(4)	2.1(4)
N(004)	23.0(5)	15.5(5)	12.2(5)	-3.6(4)	-1.4(4)	1.6(4)
C(005)	14.8(6)	17.0(6)	17.1(6)	-0.5(5)	5.2(4)	-1.4(4)
C(006)	16.2(5)	17.3(6)	10.9(5)	-0.6(4)	2.3(4)	-3.9(4)
C(007)	20.0(6)	15.8(6)	14.2(6)	-2.0(5)	0.8(5)	-0.5(5)
C(008)	17.6(6)	15.7(6)	11.2(5)	-0.5(4)	2.6(4)	-3.0(4)
C(009)	17.9(6)	14.8(6)	11.2(5)	-2.1(4)	2.7(4)	-2.0(4)
C(00A)	17.1(6)	19.2(6)	14.9(6)	-5.6(5)	4.3(5)	0.7(5)
C(00B)	17.9(6)	15.4(6)	12.7(5)	-2.3(4)	1.4(4)	-0.2(5)
C(00C)	18.0(6)	18.0(6)	12.7(5)	0.8(4)	1.8(4)	-1.9(5)
C(00D)	17.6(6)	21.9(6)	16.3(6)	1.5(5)	5.0(5)	1.9(5)
C(00E)	19.9(6)	17.9(6)	23.0(6)	-7.0(5)	8.5(5)	-1.6(5)
C(00F)	18.9(6)	28.4(7)	16.8(6)	-5.5(5)	5.8(5)	-1.1(5)
C(00G)	26.8(7)	15.4(6)	22.3(6)	-2.5(5)	10.2(5)	0.0(5)
C(00H)	20.2(6)	22.5(7)	15.5(6)	0.4(5)	3.3(5)	1.5(5)
C(00I)	20.5(6)	21.8(7)	26.1(7)	-5.9(5)	7.6(5)	-1.5(5)
C(00J)	23.2(6)	19.8(6)	26.4(7)	-7.7(5)	11.1(5)	-2.7(5)
C(00K)	30.0(7)	15.7(6)	16.2(6)	-1.1(5)	1.0(5)	-0.5(5)
C(00L)	24.5(7)	18.3(6)	25.3(7)	1.8(5)	8.6(5)	3.3(5)
C(00M)	28.0(7)	23.7(7)	36.0(8)	-13.6(6)	6.1(6)	-5.5(6)
C(00N)	48.7(9)	17.5(7)	32.3(8)	1.6(6)	12.9(7)	-0.8(6)

Table S4 Bond Lengths for compound 1.

Atom	Atom	Length/Å	Atom	Atom	Length/Å
O(001)	C(006)	1.3348(14)	C(00A)	C(00B)	1.5332(16)
O(002)	C(007)	1.2332(15)	C(00A)	C(00E)	1.4568(18)
O(003)	C(00A)	1.2313(16)	C(00B)	C(00I)	1.5436(17)
N(004)	C(007)	1.3761(16)	C(00B)	C(00K)	1.5416(17)
N(004)	C(008)	1.3992(15)	C(00D)	C(00F)	1.3831(18)
C(005)	C(00C)	1.4631(17)	C(00E)	C(00G)	1.3455(19)
C(005)	C(00D)	1.4002(17)	C(00E)	C(00M)	1.4976(18)
C(005)	C(00H)	1.4066(17)	C(00F)	C(00J)	1.3849(19)
C(006)	C(008)	1.4743(16)	C(00G)	C(00K)	1.5051(17)
C(006)	C(009)	1.3548(17)	C(00G)	C(00N)	1.4903(19)
C(007)	C(009)	1.4734(16)	C(00H)	C(00L)	1.3872(18)
C(008)	C(00C)	1.3441(17)	C(00J)	C(00L)	1.3905(19)
C(009)	C(00B)	1.5085(16)			

Table S5 Bond Angles for compound 1.

Atom	Atom	Atom	Angle/°	Atom	Atom	Atom	Angle/°
C(007)	N(004)	C(008)	110.43(10)	C(009)	C(00B)	C(00A)	114.73(10)
C(00D)	C(005)	C(00C)	123.96(11)	C(009)	C(00B)	C(00I)	109.65(10)
C(00D)	C(005)	C(00H)	118.03(11)	C(009)	C(00B)	C(00K)	114.13(10)
C(00H)	C(005)	C(00C)	117.99(11)	C(00A)	C(00B)	C(00I)	105.76(10)
O(001)	C(006)	C(008)	118.49(10)	C(00A)	C(00B)	C(00K)	102.05(10)
O(001)	C(006)	C(009)	131.11(11)	C(00K)	C(00B)	C(00I)	110.00(10)
C(009)	C(006)	C(008)	110.39(10)	C(008)	C(00C)	C(005)	130.11(11)
O(002)	C(007)	N(004)	124.38(11)	C(00F)	C(00D)	C(005)	120.58(12)
O(002)	C(007)	C(009)	127.51(11)	C(00A)	C(00E)	C(00M)	121.51(12)
N(004)	C(007)	C(009)	108.10(10)	C(00G)	C(00E)	C(00A)	108.97(11)
N(004)	C(008)	C(006)	104.75(10)	C(00G)	C(00E)	C(00M)	129.50(13)
C(00C)	C(008)	N(004)	131.18(11)	C(00D)	C(00F)	C(00J)	120.98(12)
C(00C)	C(008)	C(006)	124.03(11)	C(00E)	C(00G)	C(00K)	112.30(11)
C(006)	C(009)	C(007)	106.00(10)	C(00E)	C(00G)	C(00N)	127.89(12)
C(006)	C(009)	C(00B)	131.74(11)	C(00N)	C(00G)	C(00K)	119.80(12)
C(007)	C(009)	C(00B)	121.85(10)	C(00L)	C(00H)	C(005)	120.74(12)
O(003)	C(00A)	C(00B)	125.04(11)	C(00F)	C(00J)	C(00L)	119.18(12)
O(003)	C(00A)	C(00E)	125.46(11)	C(00G)	C(00K)	C(00B)	105.00(10)
C(00E)	C(00A)	C(00B)	109.38(10)	C(00H)	C(00L)	C(00J)	120.33(12)

Table S6 Hydrogen Atom Coordinates (Å×10^4) and Isotropic Displacement Parameters (Å^2×10^3) for compound 1.

Atom	*x*	*y*	*z*	U(eq)
H(001)	8390	6042	4546	29
H(004)	6228	4527	811	21
H(00C)	9110	4059	3442	20
H(00D)	7570	3536	411	22
H(00F)	7628	2126	-457	25
H(00H)	9714	2480	3518	23
H(00A)	4163	6821	2892	34
H(00B)	4745	7654	3714	34
H(00E)	5205	6655	4131	34
H(00J)	8665	879	646	27
H(00G)	6386	7634	1254	26
H(00I)	4943	7918	1596	26
H(00L)	9762	1072	2631	27
H(00K)	8328	9182	5094	44
H(00M)	8239	9853	4034	44
H(00N)	9471	9120	4331	44
H(00O)	6883	10072	2226	48
H(00P)	5374	9698	1546	48
H(00Q)	6739	9527	1048	48

Experimental

Single crystals of $C_{76}H_{76}N_4O_{12}$ **[20220623-HXS-H1]** were **[]**. A suitable crystal was selected and **[]** on a **XtaLAB Synergy, Dualflex, HyPix** diffractometer. The crystal was kept at 103(6) K during data collection. Using Olex2, the structure was solved with the ShelXT structure solution program using Intrinsic Phasing and refined with the ShelXL refinement package using Least Squares minimisation.

Crystal structure determination of [20220623-HXS-H1]

Crystal Data for $C_{76}H_{76}N_4O_{12}$ (M =1229.34 g/mol): monoclinic, space group P2$_1$/c (no. 14), a = 9.54190(10) Å, b = 14.6110(3) Å, c = 11.6889(2) Å, β = 103.786(2)°, V = 1582.68(5) Å^3, Z = 1, T = 103(6) K, μ(CuKα) = 0.709 mm^{-1}, $Dcalc$ = 1.290 g/cm^3, 10965 reflections measured (9.544° ≤ 2Θ ≤ 151.32°), 3116 unique (R_{int} = 0.0274, R_{sigma} = 0.0238) which were used in all calculations. The final R_1 was 0.0381 (I > 2σ(I)) and wR_2 was 0.0995 (all data).

Refinement model description

Number of restraints - 0, number of constraints - unknown.

Details:

1. Fixed Uiso

At 1.2 times of:

All C(H) groups, All C(H,H) groups, All N(H) groups

At 1.5 times of:
All C(H,H,H) groups, All O(H) groups
2.a Secondary CH2 refined with riding coordinates:
C00K(H00G,H00I)
2.b Aromatic/amide H refined with riding coordinates:
N004(H004), C00C(H00C), C00D(H00D), C00F(H00F), C00H(H00H), C00J(H00J), C00L(H00L)
2.c Idealised Me refined as rotating group:
C00I(H00A,H00B,H00E), C00M(H00K,H00M,H00N), C00N(H00O,H00P,H00Q)
2.d Idealised tetrahedral OH refined as rotating group:
O001(H001)

This report has been created with Olex2, compiled on 2018.05.29 svn.r3508 for OlexSys. Please let us know if there are any errors or if you would like to have additional features.
